# From Farm to Fork: Persistence of Clinically Relevant Multidrug-Resistant and Copper-Tolerant Klebsiella pneumoniae Long after Colistin Withdrawal in Poultry Production

**DOI:** 10.1128/spectrum.01386-23

**Published:** 2023-07-10

**Authors:** Joana Mourão, Marisa Ribeiro-Almeida, Carla Novais, Mafalda Magalhães, Andreia Rebelo, Sofia Ribeiro, Luísa Peixe, Ângela Novais, Patrícia Antunes

**Affiliations:** a UCIBIO—Applied Molecular Biosciences Unit, REQUIMTE, Laboratory of Microbiology, Department of Biological Sciences, Faculty of Pharmacy, University of Porto, Porto, Portugal; b Associate Laboratory i4HB—Institute for Health and Bioeconomy, Faculty of Pharmacy, University of Porto, Porto, Portugal; c Center for Innovative Biomedicine and Biotechnology (CIBB), University of Coimbra, Coimbra, Portugal; d School of Medicine and Biomedical Sciences, University of Porto (ICBAS-UP), Porto, Portugal; e Faculty of Nutrition and Food Sciences, University of Porto, Porto, Portugal; f ESS, Polytechnic of Porto, Porto, Portugal; USDA-ARS

**Keywords:** chicken meat, poultry sector, copper feed supplementation, antibiotic resistance, IncF plasmids, One Health, food safety

## Abstract

Concerns about colistin-resistant bacteria in animal food-environmental-human ecosystems prompted the poultry sector to implement colistin restrictions and explore alternative trace metals/copper feed supplementation. The impact of these strategies on the selection and persistence of colistin-resistant Klebsiella pneumoniae in the whole poultry production chain needs clarification. We assessed colistin-resistant and copper-tolerant K. pneumoniae occurrence in chickens raised with inorganic and organic copper formulas from 1-day-old chicks to meat (7 farms from 2019 to 2020), after long-term colistin withdrawal (>2 years). Clonal diversity and K. pneumoniae adaptive features were characterized by cultural, molecular, and whole-genome-sequencing (WGS) approaches. Most chicken flocks (75%) carried K. pneumoniae at early and preslaughter stages, with a significant decrease (*P* < 0.05) in meat batches (17%) and sporadic water/feed contamination. High rates (>50%) of colistin-resistant/*mcr*-negative K. pneumoniae were observed among fecal samples, independently of feed. Most samples carried multidrug-resistant (90%) and copper-tolerant (81%; *silA* and *pcoD* positive and with a MIC_CuSO4_ of ≥16 mM) isolates. WGS revealed accumulation of colistin resistance-associated mutations and F type multireplicon plasmids carrying antibiotic resistance and metal/copper tolerance genes. The K. pneumoniae population was polyclonal, with various lineages dispersed throughout poultry production. ST15-KL19, ST15-KL146, and ST392-KL27 and IncF plasmids were similar to those from global human clinical isolates, suggesting chicken production as a reservoir/source of clinically relevant K. pneumoniae lineages and genes with potential risk to humans through food and/or environmental exposure. Despite the limited *mcr* spread due to the long-term colistin ban, this action was ineffective in controlling colistin-resistant/*mcr*-negative K. pneumoniae, regardless of feed. This study provides crucial insights into the persistence of clinically relevant K. pneumoniae in the poultry production chain and highlights the need for continued surveillance and proactive food safety actions within a One Health perspective.

**IMPORTANCE** The spread of bacteria resistant to last-resort antibiotics such as colistin throughout the food chain is a serious concern for public health. The poultry sector has responded by restricting colistin use and exploring alternative trace metals/copper feed supplements. However, it is unclear how and to which extent these changes impact the selection and persistence of clinically relevant Klebsiella pneumoniae throughout the poultry chain. We found a high occurrence of copper-tolerant and colistin-resistant/*mcr*-negative K. pneumoniae in chicken flocks, regardless of inorganic and organic copper formulas use and a long-term colistin ban. Despite the high K. pneumoniae isolate diversity, the occurrence of identical lineages and plasmids across samples and/or clinical isolates suggests poultry as a potential source of human K. pneumoniae exposure. This study highlights the need for continued surveillance and proactive farm-to-fork actions to mitigate the risks to public health, relevant for stakeholders involved in the food industry and policymakers tasked with regulating food safety.

## INTRODUCTION

A major public health concern has been attributed to colistin-resistant bacteria, particularly those carrying mobilized colistin resistance (*mcr*) genes, due to implications for human, animal, and environmental health (1, 2). The wide use of colistin for decades in veterinary medicine imposed global restrictions on food animal production (3–5). Colistin in the European Union is categorized as “B—Restrict,” which is reserved for clinical infections for which no alternatives are available, in compliance with 2022 regulations that ban routine antibiotic use in farming. Additionally, colistin restrictions align with the 2030 European goal of reducing farm animal antibiotic sales by 50%, with consequential effects on diverse environmental compartments (6–10). Diverse studies revealed a decreasing occurrence of *mcr*-carrying bacteria (mainly Escherichia coli and Klebsiella pneumoniae) in farms shortly after colistin restrictions (11, 12), whereas the persistence of colistin-resistant bacteria by mechanisms other than *mcr* is unknown. Furthermore, there are no studies evaluating the long-term impact of colistin restriction on the occurrence and diversity of colistin-resistant bacteria throughout food animal production environments and/or considering farm-level variation or food chain practices (4, 11). Studies on intensive poultry meat systems are crucial for informing strategies to reduce or replace antibiotics, given the European Union's prominent position as one of the top four global chicken meat producers and the increasing consumer demand for “greener” antibiotic-free food products (13–15).

Along the same line as colistin restriction is the development of nutritional alternatives to improve poultry health and growth, including the use of copper (Cu) in feeds (3, 15–17). Copper is a heavy metal widely used in poultry feed due to its requirement in many physiologic processes, immune-stimulating actions, and antimicrobial activity in the gut (15, 18, 19). In current commercial practice, inorganic trace mineral formulation feeds (ITMF) are supplemented with inorganic sources of Cu (e.g., Cu sulfate and Cu oxide) at high levels (<25 mg/kg; accordingly to EU Regulation 2018/1039), leading to potential environmental accumulation (20–22). Alternatively, organic trace mineral feed (OTMF) supplements (e.g., Cu chelated with amino acids, peptides, or proteins) have been associated with a higher Cu bioavailability in the digestive tract than that with ITMF and an animal performance improvement, with reduced fecal excretion and environmental release expected (19–23). However, the effect of different Cu-supplemented feeds on gastrointestinal microbiota composition is still unclear and needs to be further explored by experimental studies (18–20, 24). In addition, a link has been proposed between feed supplementation with Cu (and other metals) and the selection of antibiotic-resistant bacteria (24, 25). However, the impact of variable feed formulations (OTMF versus ITMF) on poultry gut bacteria, particularly in species often associated with colistin resistance, and throughout the whole food production chain has been poorly explored.

K. pneumoniae has been frequently associated with colistin resistance and *mcr* carriage in the clinical setting, but its occurrence in the animal food-environmental interface is underexplored (26). Besides, we have previously shown the colocalization of metal/Cu operons with the *mcr-1* gene in plasmids from K. pneumoniae isolated from chicken meat batches shortly after colistin withdrawal (12), suggesting a Cu coselection effect for the persistence of colistin resistance. In fact, K. pneumoniae is a versatile species with a natural ability to acquire antibiotic and metal resistance genes, enabling their survival and circulation among animal-environment-human ecosystems (27–29). However, it remains unclear how and to what extent restriction/replacement of colistin interventions in poultry production impacts the selection and persistence of colistin-resistant K. pneumoniae (ColR-Kp) and, eventually, clones with a potential risk of foodborne and/or environmental transmission.

We used a One Health approach by spanning the poultry production chain (from 1-day-old chicks to meat) to better understand the impact of the long-term colistin ban on the occurrence of colistin-resistant and Cu-tolerant K. pneumoniae and its association with ITMF and OTMF feed formulations. We applied whole-genome sequencing (WGS) to characterize these isolates in terms of clonal diversity, antibiotic resistance, metal tolerance, and virulence determinants to understand the risks that exist in the poultry environment.

## RESULTS

### Occurrence of K. pneumoniae and colistin-resistant K. pneumoniae by samples.

**FIG 1:**
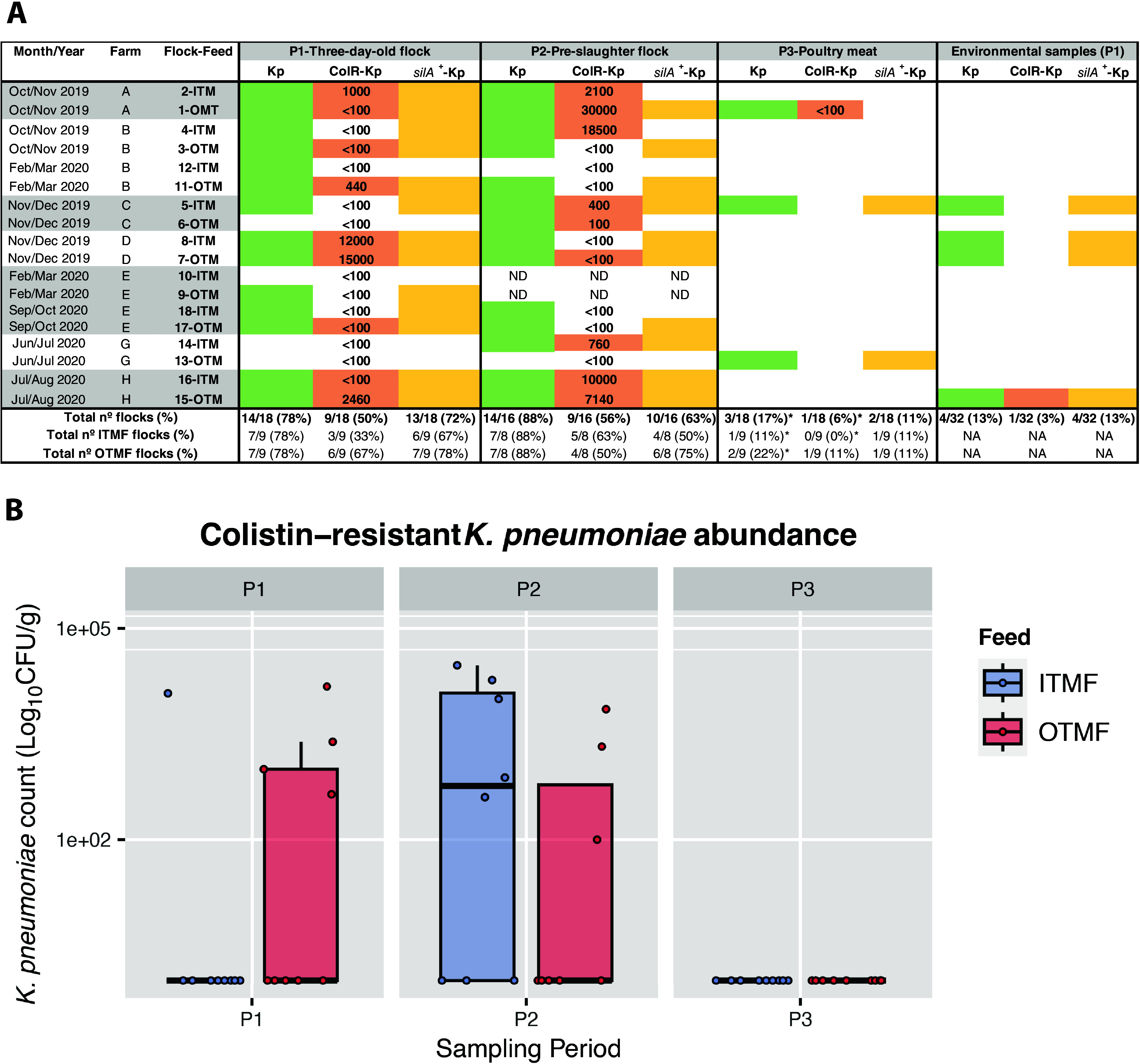
(A) Occurrence of K. pneumoniae (Kp-green), colistin-resistant K. pneumoniae (ColR-Kp-orange; the numbers represent an estimate [CFU/g] of ColR-Kp), and *silA*-carrying K. pneumoniae (silA^+^-Kp - yellow) among flocks (feces, P1 and P2) and chicken meat (P3) by type of feed (ITMF or OTMF). Environmental samples 5, 7, and 8 were from feed, and sample 15 corresponds to water. *, *P* < 0.05 (Fisher’s exact test), when P2 is compared with P3. ND, not determined; NA, not applicable. (B) Box plot of colistin-resistant K. pneumoniae counts among flocks (feces, P1 and P2) and chicken meat (P3) by type of feed (ITMF or OTMF). *P* > 0.05 was calculated using the Wilcoxon test for a comparison of P1 with P2 (both feeds). The median is represented by a black horizontal line, and blue (ITMF) or red (OTMF) data points represent the counts in each sample.

Our culture approach detected K. pneumoniae in 42% (*n* = 35/84) of the total tested samples within the poultry production chain. A high occurrence was found among fecal samples (82%; 78% [14/18 flocks] at the P1 stage [when chicks were 2 to 3 days old] and 88% [14/16 flocks] at the P2 stage [the day before slaughter]), corresponding in all but two of the flocks and all farms tested. However, a significant decrease in occurrence was detected between stage P2 and chicken meat batches (17% [3/18] at stage P3 [after slaughter], corresponding to flocks from 3 farms) (*P* < 0.05) (Fig. 1A). Only two flocks (2 farms) had K. pneumoniae isolates in all three stages (P1, P2, and P3), while the remaining ones were found in stage P1 and P2 (10 flocks) (75% [12/16 flocks] at P1 and P2 and/or P3) or only in stage P1 (1 flock), P2 (2 flocks), or P3 (1 flock). Similar occurrence rates were observed between OTMF and ITMF samples at each of the three sampling stages (*P* > 0.05) (Fig. 1A). Regarding poultry house environmental samples, three feed samples (2 farms) and one water sample (another farm) were contaminated with K. pneumoniae.

The ColR-Kp isolates were recovered from all farms and sample types, corresponding to 57% (20/35) of K. pneumoniae-positive samples. Most were fecal samples at stage P1 (50% [9/18 flocks]; 5 farms) and P2 (56% [9/16 flocks]; 6 farms), with no significant difference by feed type at the two stages (*P* > 0.05) (Fig. 1A). We noticed in the follow-up of flocks that ColR-Kp occurrence in chicken feces was maintained (*n* = 5 flocks) or increased (*n* = 4 flocks) from stage P1 to P2, with no significant difference in the ColR-Kp levels between the two stages (*P* > 0.05) (Fig. 1B). ColR-Kp was detected in only one of the three chicken meat batches contaminated with K. pneumoniae obtained from a farm which was also positive at stage P1 and P2. Regarding environmental samples, ColR-Kp was found in one water sample from one farm, also positive at stage P1 and P2. All isolates were negative for the screening of *mcr* genes but presented MICs for colistin ranging between 4 and ≥16 mg/L, which is compatible with the presence of other acquired resistance mechanisms (for results, see Whole-genome analysis of *K. pneumoniae* poultry-associated isolates).

### Diversity of K. pneumoniae and colistin-resistant K. pneumoniae isolates.

**FIG 2:**
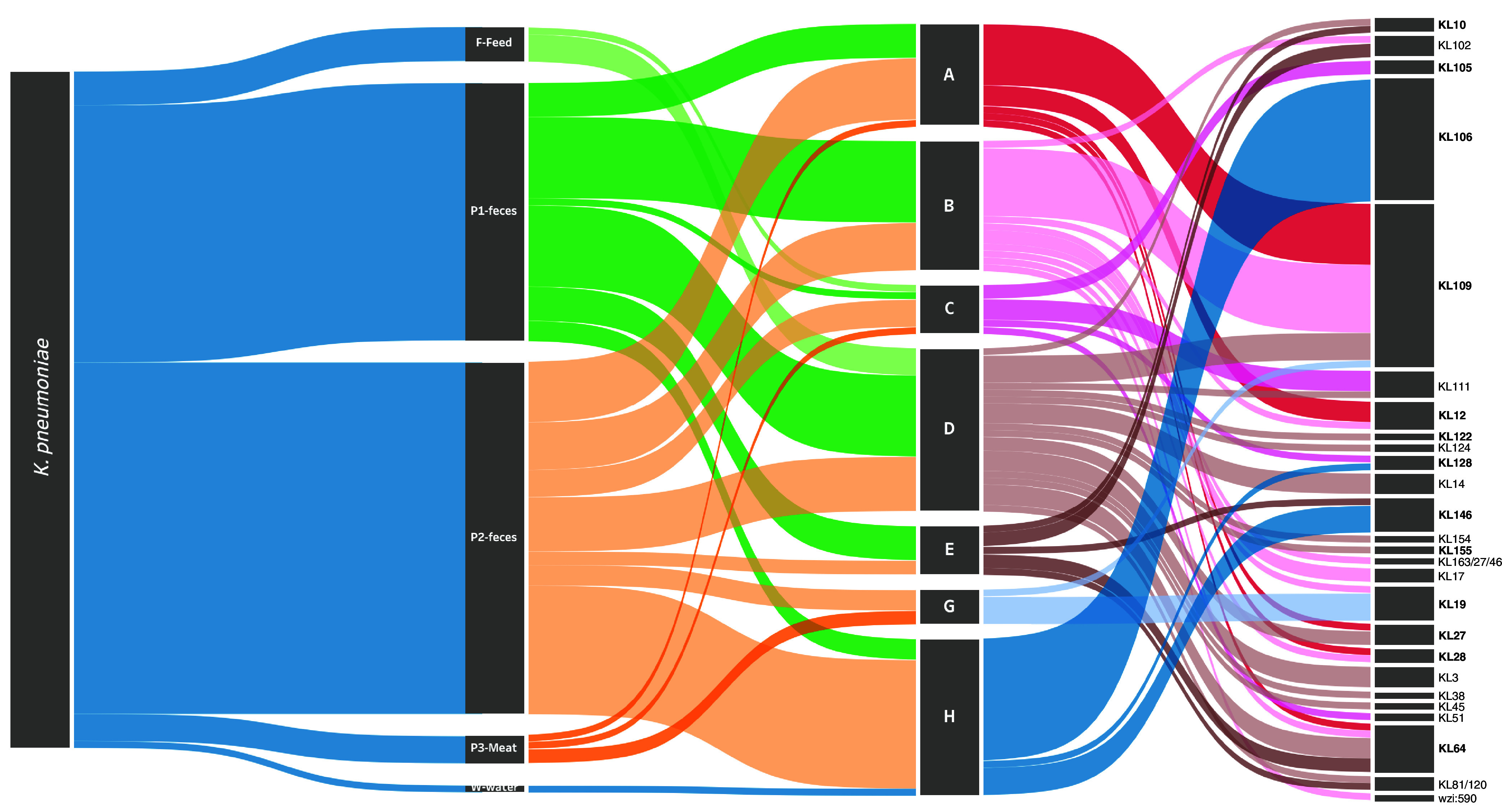
Sankey diagram representing, from left to right, the occurrence and diversity of K. pneumoniae isolates by sample, farm, and KL type(s). The width of each connection is proportional to the number of positive hits. The KL type(s) comprising colistin-resistant isolates are indicated in bold. The Sankey diagram was generated using Tableau Desktop 2021.4 (https://www.tableau.com/).

We recovered 100 *K. pneumoniae* isolates (50 from OTMF and 50 from ITMF flocks), corresponding to 35 positive samples. Of these isolates, 56 were colistin resistant, corresponding to 20 positive samples. Most isolates (90%) were recovered from P1 and P2 fecal samples (16 flocks, all farms) (Fig. 2). K. pneumoniae isolates were grouped into 26 capsular locus (KL) - types, with 12 dispersed in more than one farm (Fig. 2; see Table S1 in the supplemental material). Two KL type(s) represented 40% (*n* = 42) of isolates, independently of feed, and included colistin-resistant isolates KL109, which was widely dispersed (*n* = 24; 4 farms; P1, P2, and P3; farm B—flock 4 and flock 11 over 4 months), and, in contrast, KL106, which was present only in one farm (*n* = 18; water, P1 and P2) (Fig. 2). Although less frequent, other KL type(s) were shared by different farms or stages: KL12 (2 farms; P1), KL19 (2 farms; P1, P2, and P3), KL64 (4 farms; P1 and P2), KL111 (2 farms; feed, P1 and P3), KL27 (2 farms; feed and P1) and KL146 (2 farms; P2) (Fig. 2; Table S1). There were 22 different KL type(s) observed among the colistin-susceptible isolates (*n* = 44), while the colistin-resistant isolates (*n* = 56) exhibited 13 different KL type(s). Additionally, 9 KL type(s) were found to be present in both groups.

### Antibiotic susceptibility of K. pneumoniae recovered from poultry production.

**FIG 3:**
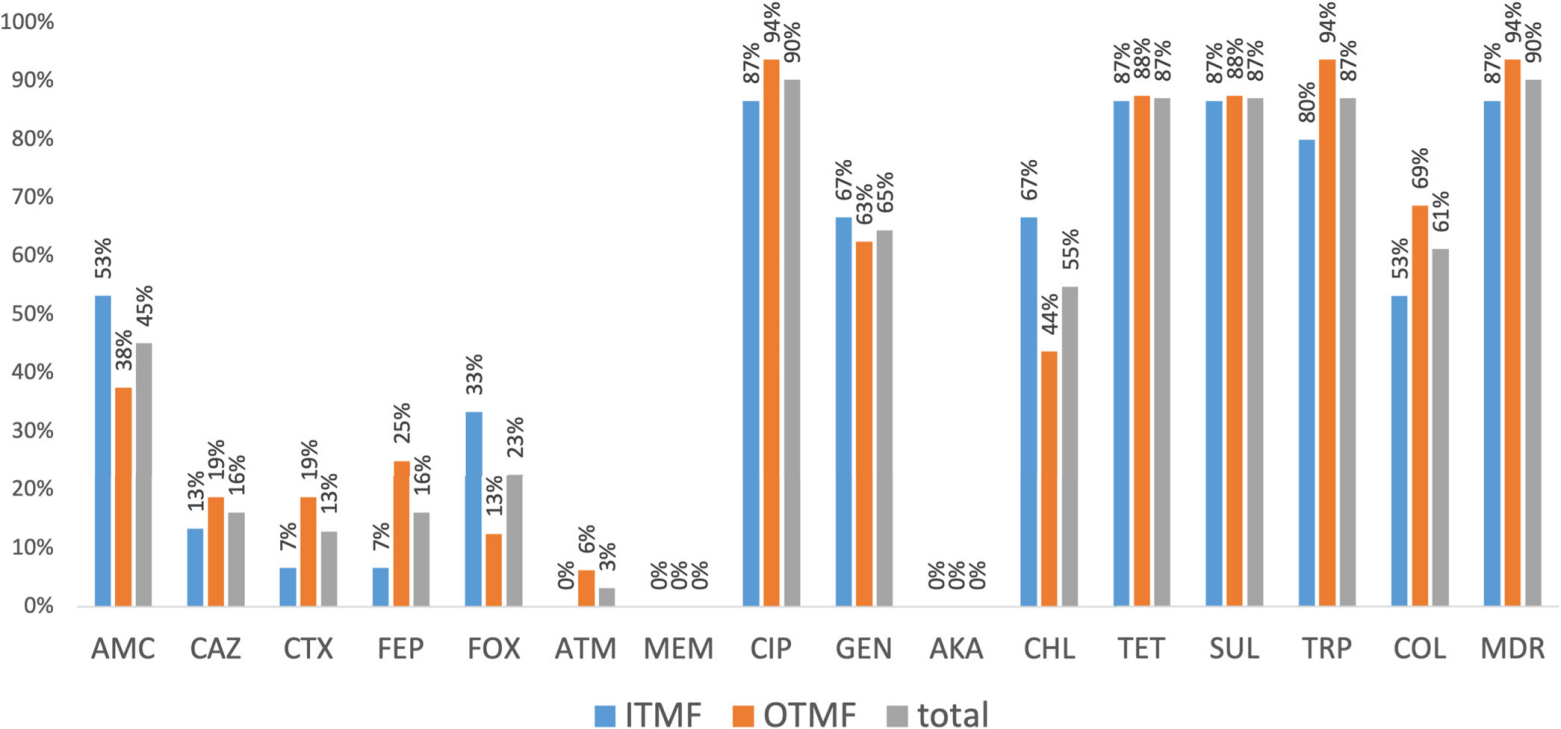
Occurrence of antibiotic-resistant K. pneumoniae isolates among positive poultry samples (P1, P2, and P3) by type of feed (ITMF - blue and OTMF - orange). *P* > 0.05 (Fisher’s exact test). AMC, amoxicillin-clavulanic acid; CAZ, ceftazidime; CTX, cefotaxime; FEP, cefepime; FOX, cefoxitin; ATM, aztreonam; MEM, meropenem; CIP, ciprofloxacin; GEN, gentamicin; AKA, amikacin; CHL, chloramphenicol; TET, tetracycline; SUL, sulfonamides; TRP, trimethoprim; COL, colistin; MDR, multidrug resistance.

Considering poultry and meat samples with K. pneumoniae (*n* = 31), antibiotic-resistant isolates were observed in all but one sample. Most of these (90%, *n* = 28/31) carried multidrug-resistant (MDR) isolates, corresponding to all farms and all but two flocks. Similar MDR and antibiotic resistance rates per sample were observed between both types of feed (*P* > 0.05) (Fig. 3). More than 50% of the samples presented at least one K. pneumoniae isolate with decreased susceptibility to ciprofloxacin (90%, *n* = 28/31) or resistance to tetracycline, sulfonamides, or trimethoprim (87%, *n* = 27/31 each), gentamicin (65%, *n* = 20/31), colistin (61%, *n* = 19/31), or chloramphenicol (55%, *n* = 17/31) (Fig. 3). Regarding the environmental samples with K. pneumoniae (feed [*n* = 5; 3 samples] and water [*n* = 1; 1 sample]) all carried MDR isolates. More than 15% of samples (including feces, chicken meat, and water) from diverse farms/flocks presented at least one isolate that was resistant to extended-spectrum cephalosporins.

K. pneumoniae isolates resistant to colistin (*n* = 56/100) were mainly recovered in Simmons citrate agar with 1% inositol (SCAi) medium supplemented with this antibiotic, with or without the previous enrichment step (*n* = 24 and *n* = 30, respectively) (Table 1). High resistance rates to ciprofloxacin (86%), tetracycline (79%), sulfonamides (74%), and trimethoprim (75%) were observed as well as MDR (80%) independently of the selection strategy or colistin phenotype (Table 1). The MDR isolates belonged to different KL type(s) (*n* = 23). Among these isolates, the most frequent phenotypes were resistance to ciprofloxacin, tetracycline, sulfonamides, and trimethoprim (*n* = 62 isolates [62%]; 18 KL type(s)). Additionally, some of these isolates showed resistance to colistin (*n* = 33 isolates; 9 KL type(s)) and/or extended-spectrum cephalosporins (*n* = 26 isolates; 7 KL type(s)) (Table S1).

**TABLE 1:** Antimicrobial susceptibility of K. pneumoniae isolates (*n* = 100), including colistin-resistant K. pneumoniae isolates (*n* = 56) recovered from poultry production samples

Isolate selection	K. pneumoniae (no. of isolates; no. of samples)	Antimicrobial resistance [no. of isolates (%)]^*a*^																
		COL	AMC	CAZ	CTX	FEP	FOX	ATM	MEM	CIP	AKA	GEN	CHL	TET	SUL	TRP	MDR	*silA* and *pcoD* positive
SCAi without enrichment/direct	COL-R (1; 1)	1	1	1	0	0	1	0	0	1	0	1	1	0	1	1	1	2
	COL-S (6; 5)	0	5	0	0	0	1	0	0	6	0	2	4	6	6	6	6	5
	Total (7; 6)	1 (14)	6 (86)	1 (14)	0	0	2 (29)	0	0	7 (100)	0	3 (43)	5 (71)	6 (86)	7 (100)	7 (100)	7 (100)	7 (100)
SCAi with enrichment/BPW	COL-R (1; 1)	1	0	0	0	0	0	0	0	1	0	0	0	1	0	0	0	0
	COL-S (3; 3)	0	0	0	0	0	0	0	0	3	0	1	2	3	3	3	3	3
	Total (4; 4)	1 (25)	0	0	0	0	0	0	0	4 (100)	0	1 (25)	2 (50)	4 (100)	3 (75)	3 (75)	3 (75)	3 (75)
SCAi plus COL (3.5 μg/mL) without enrichment/direct	COL-R (30; 13)	30	13	9	0	2	13	0	0	28	0	15	14	23	22	21	24	16
	COL-S (21; 18)	0	3	0	1	1	2	0	0	16	0	5	8	14	11	12	15	17
	Total (51; 24)	30 (59)	16 (31)	9 (18)	1 (2)	3 (6)	15 (29)	0	0	44 (86)	0	20 (39)	22 (43)	37 (73)	33 (65)	33 (65)	39 (76)	33 (65)
SCAi plus COL (3.5 μg/mL) with enrichment/BPW	COL-R (24; 14)	24	10	7	2	3	6	0	0	20	0	16	10	21	21	21	21	13
	COL-S (14; 13)	0	3	2	2	1	1	1	0	11	0	4	3	11	10	11	10	11
	Total (38; 21)	24 (63)	13 (34)	9 (24)	4 (11)	4 (11)	7 (18)	1 (3)	0	31 (82)	0	20 (53)	13 (34)	32 (84)	31 (82)	32 (84)	31 (82)	24 (63)
All isolates	Total (100; 35)	56 (56)	35 (35)	19 (19)	5 (5)	7 (7)	24 (24)	1 (1)	0 (0)	86 (86)	0 (0)	44 (44)	42 (42)	79 (79)	74 (74)	75 (75)	80 (80)	67 (67)

a COL, colistin; COL-R, colistin-resistant K. pneumoniae; COL-S, colistin-susceptible K. pneumoniae; AMC, amoxicillin-clavulanic acid; CAZ, ceftazidime; CTX, cefotaxime; FEP, cefepime; FOX, cefoxitin; ATM, aztreonam; MEM, meropenem; CIP, ciprofloxacin; AKA, amikacin; GEN, gentamicin; CHL, chloramphenicol; TET, tetracycline; SUL, sulfonamides; TRP, trimethoprim; MDR, multidrug resistance; SCAi, Simmons citrate agar with 1% inositol; BPW, buffered peptone water.

### Copper tolerance of K. pneumoniae recovered from poultry production.

**FIG 4:**
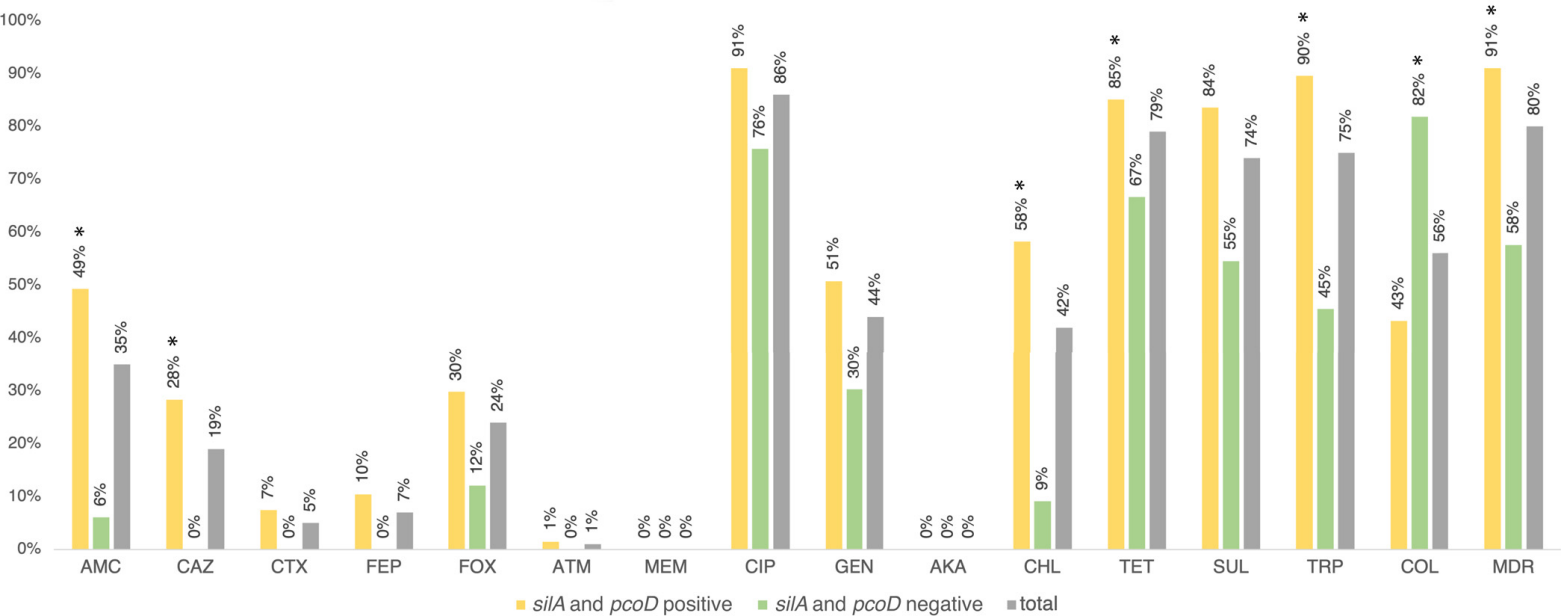
Percentage of antibiotic resistance detected among *silA* and *pcoD* positive - yellow (*n* = 67) and *silA* and *pcoD* negative - light green (*n* = 33) K. pneumoniae isolates. *, *P* < 0.05 (Fisher’s exact test). AMC, amoxicillin-clavulanic acid; CAZ, ceftazidime; CTX, cefotaxime; FEP, cefepime; FOX, cefoxitin; ATM, aztreonam; MEM, meropenem; CIP, ciprofloxacin; GEN, gentamicin; AKA, amikacin; CHL, chloramphenicol; TET, tetracycline; SUL, sulfonamides; TRP, trimethoprim; COL, colistin; MDR, multidrug resistance.

The copper tolerance genes *silA* and *pcoD* were observed in isolates dispersed in all farms, most flocks (88%, *n* = 14/16), and poultry samples with K. pneumoniae (81%, *n* = 25/31) (Fig. 1A). Similar rates were observed between ITMF and OTMF samples among different sampling periods (*P* > 0.05). The *silA* and *pcoD* genes were detected in 67% (*n* = 67/100) of the K. pneumoniae isolates, regardless of their origin by feed source (70%, *n* = 35/50 ITMF versus 64%, *n* = 32/50 OTMF) or farm stage (*n* = 28 at P1 versus *n* = 30 at P2). The *silA* and *pcoD* positive isolates were also detected in chicken meat (*n* = 3; 2 samples at P3) and environmental samples (feed [*n* = 5; 3 samples] and water [*n* = 1; 1 sample]). The *silA* and *pcoD* positive isolates belonged to 25 of 26 KL type(s), compared to the *silA* and *pcoD* negative isolates, which belonged to only 9 KL type(s). Regarding antibiotic susceptibility, MDR was mostly found in *silA-* and *pcoD*-positive K. pneumoniae isolates (91%, *n* = 61/67 compared to 58%, *n* = 19/33 of isolates without *silA* and *pcoD* [*P* < 0.05]), with those also being more resistant to amoxicillin-clavulanic acid, ceftazidime (including extended-spectrum β-lactamase [ESBL] producers), chloramphenicol, tetracycline, and trimethoprim (*P* < 0.05) (Fig. 4). The correlation between colistin resistance and the absence of *silA* and *pcoD* can be explained by the predominance of the colistin-resistant KL109. More specifically, of the 33 isolates that lacked *silA* and *pcoD*, 23 of them corresponded to K type, which exhibited colistin resistance (Fig. 4; Table S1).

Copper phenotypic assays were performed in 85% of K. pneumoniae isolates carrying or not carrying copper tolerance genes representative of different farms, flocks, KL type(s), and antibiotic resistance profiles (Table 2). All K. pneumoniae isolates carrying the *silA* and *pcoD* genes (100%, *n* = 53/53) exhibited MICs to CuSO_4_ of 16 to 32 mM (CuT phenotype, MIC_CuSO4_ ≥ 16 mM). These data contrast with those of most K. pneumoniae isolates without acquired copper tolerance *silA* and *pcoD* genes (*n* = 30/32), showing MICs to CuSO_4_ of 2 to 12 mM (Table 2). The MIC_CuSO4_ distributions were similar for isolates of the two feed sources (ITMF and OTMF).

**TABLE 2:** CuSO_4_ MICs in anaerobiosis for K. pneumoniae isolates (*n* = 85) by poultry feed type

Feed	*silA* and *pcoD* gene	Total no. of isolates^*a*^	No. of isolates with indicated MIC (mM)^*b*^											
			0.5	1	2	4	8	12	16	20	24	28	32	36
ITMF	+	29							7	7	12	3		
	−	14				1	4	8	1					
OTMF	+	24							4	6	12	1	1	
	−	18			1	5	1	10	1					
Total	+	53							11	13	24	4	1	
	−	32			1	6	5	18	2					

a The 85 isolates were selected to represent diverse farms, flocks, feeds, copper genotypes, and genomic backgrounds.

b E. coli ED8739 (plasmid pRJ1004 with *sil* and *pco* genes; MIC_CuSO4_, 16 to 20 mM, anaerobiosis) and Enterococcus faecium BM4105RF (negative for all genes tested; MIC_CuSO4_, 2 to 4 mM, anaerobiosis) were used as control strains in Cu assays (63). Corresponding CuSO_4_ values in milligrams per liter for the millimolar values tested are as follows: 0.5 mM, 79.80 mg/L; 1 mM, 159.61 mg/L; 2 mM, 319.22 mg/L; 4 mM, 638.44 mg/L; 8 mM, 1,276.87 mg/L; 12 mM, 1,915.308 mg/L; 16 mM, 2,553.74 mg/L; 20 mM, 3,192.18 mg/L; 24 mM, 3,830.62 mg/L; 28 mM, 4,469.05 mg/L; 32 mM, 5,107.49 mg/L; and 36 mM, 5,745.92 mg/L.

### Whole-genome analysis of K. pneumoniae poultry-associated isolates.

**FIG 5:**
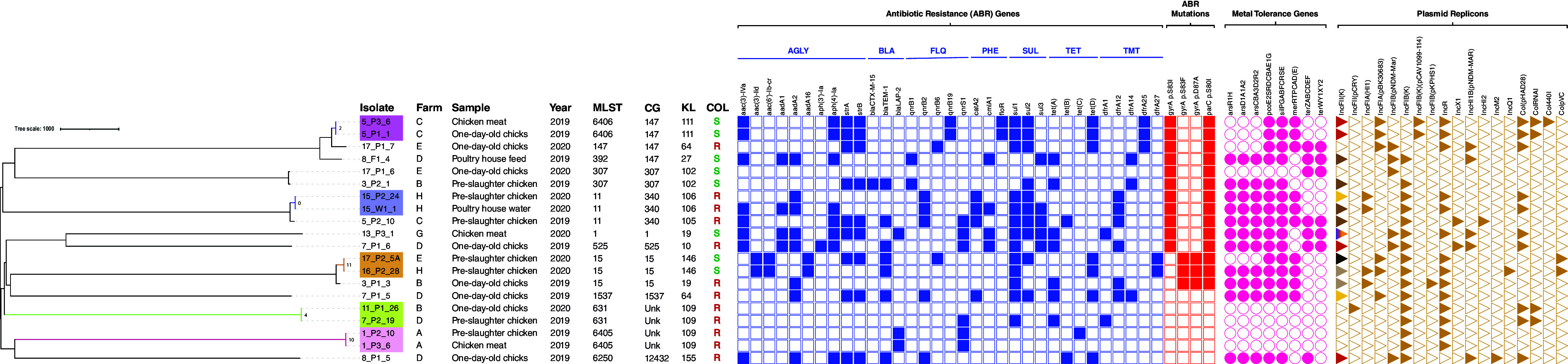
Neighbor-joining tree representing the phylogenetic relationships among the 20 K. pneumoniae genomes. The tree was constructed from the Pathogenwatch pairwise-distance matrix (i.e., based on SNPs called in 1,972 core genes). Scale bar units represent substitutions per variant site. The number of substitutions in our isolates compared to the 5 main clusters is represented in each branch. Associated metadata of all isolates were added using iTOL (https://itol.embl.de/). Each color-filled shape represents the presence of relevant antibiotic resistance, metal tolerance genes, and plasmid replicons associated with well-defined incompatibility groups. The different shades of colors represent the typing results for IncFII(K) plasmids (orange, IncFII_K2_; brown, IncFII_K4_; beige, IncFII_K5_; dark brown, IncFII_K7_; half violet/orange, IncFII_K8_; yellow, IncFII_K21-like_). Only known mutations conferring fluoroquinolone resistance are presented. Klebsiella intrinsic antibiotic resistance (*bla*_SHV-1_, *bla*_SHV-11_, *bla*_SHV-26_, *bla*_SHV-28_, *fosA*, *oxqAB*) and metal tolerance (*arsBCR*, *cusABFCRS*) genes are not represented. The isolate 17_P2_5A possesses all the genes of the *mer* operon except for *merE*, which is indicated by the use of curved brackets in merRTPCAD(E). AGLY, aminoglycosides; BLA, β-lactams; CG, clonal group; COL, colistin; FLQ, fluoroquinolones; KL, K locus; MLST, multilocus sequence typing; PHE, phenicols; SUL, sulfonamides; TET, tetracycline; TMT, trimethoprim; Unk, unknown.

**FIG 6:**
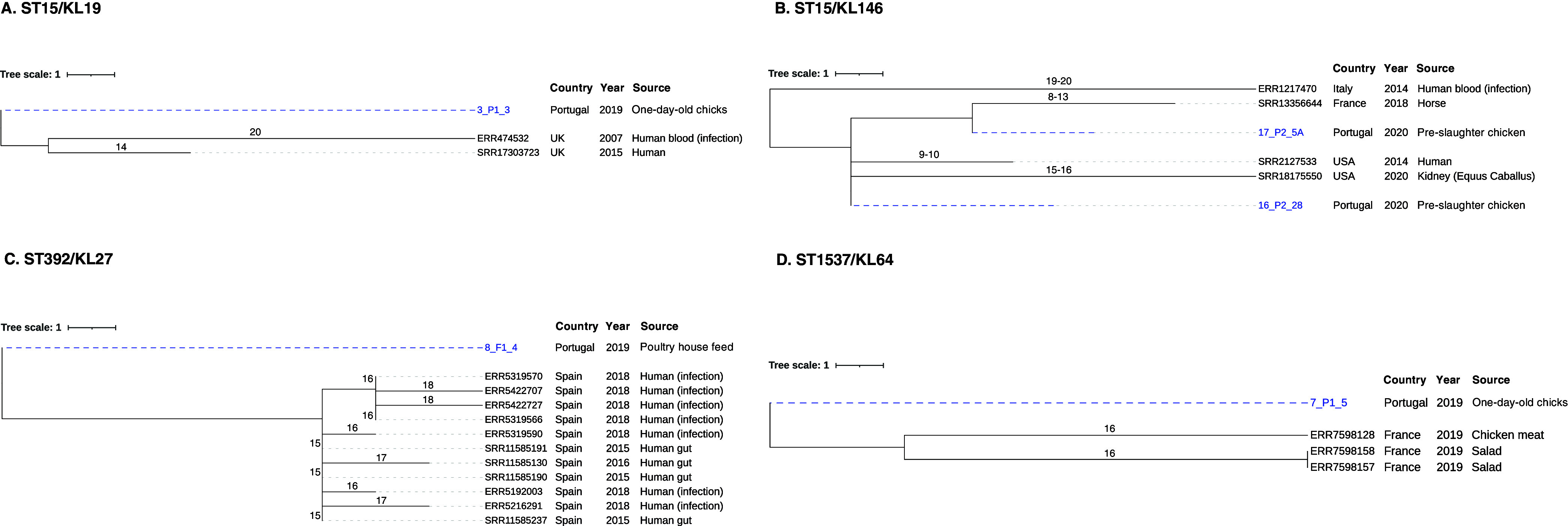
Neighbor-joining trees representing phylogenetic relationships among our K. pneumoniae genomes and those available in Pathogenwatch with fewer than 21 SNPs. (A) ST15/KL19; (B) ST15/KL146; (C) ST392/KL27; (D) ST1537/KL64. Genome selection was performed using cgMLST single linkage clustering to include the ones with fewer than 10 allele differences (threshold = 10). Then, these genomes were used to infer a neighbor-joining tree from the Pathogenwatch pairwise-distance matrix (i.e., based on SNPs called in 1,972 core genes). Scale bar units represent substitutions per variant site. The number of substitutions in our isolates and the ones available in Pathogenwatch are represented in each branch. All isolate-associated metadata (country, source of isolation, and collection date) were added using iTOL (https://itol.embl.de/).

The 20 sequenced K. pneumoniae isolates representing the most abundant KL type(s) were assigned to 12 sequence types (STs) (10 known and 2 new STs) and 14 lineages based on core genome and KL types, including globally dispersed ST11-KL105, ST15-KL19, ST147-KL64, and ST307-KL102. Based on the core genome multilocus sequence type (cgMLST) analysis and the proposed thresholds (<10 allele differences) (30–32), the K. pneumoniae isolates were clonally diverse, with 10 isolates distributed in five clusters (differing by 0 to 11 single nucleotide polymorphisms [SNPs]) (Fig. 5; Table S2). Those clusters included isolates from different sample types from the same farm, i.e., feces of poultry and derived meat (*n* = 2 ST6406/farm C and *n* = 2 ST6405/farm A), as well as water and poultry feces (*n* = 2 ST11/farm H). Also, we detected clones persisting over time in different farms (*n* = 2 ST15/farms E and H; *n* = 2 ST631/farms B and D). The phylogenetic relationship of our genomes with others from different sources, regions, and time frames available at Pathogenwatch was explored (Fig. 6), revealing fewer than 10 allele differences in all but four of the lineages (ST147, ST525, ST631, ST6405). Of note, isolates belonging to ST15-KL19, ST15-KL146, ST392-KL27, and ST1537-KL64 lineages revealed <21 SNPs with genomes from diverse origins (Fig. 6) (a threshold recently proposed for K. pneumoniae transmission in health care settings) (33). ST15 isolates were linked to genomes associated with human infections in the United Kingdom (ST15-KL19) and human infections and horses in Italy, France, and the United States (ST15-KL146). The ST392-KL27 isolate was linked to genomes from human infections and/or colonization in Spain, while ST1537-KL64 was detected in food products (chicken meat and salads) in France (Fig. 6).

**FIG 7:**
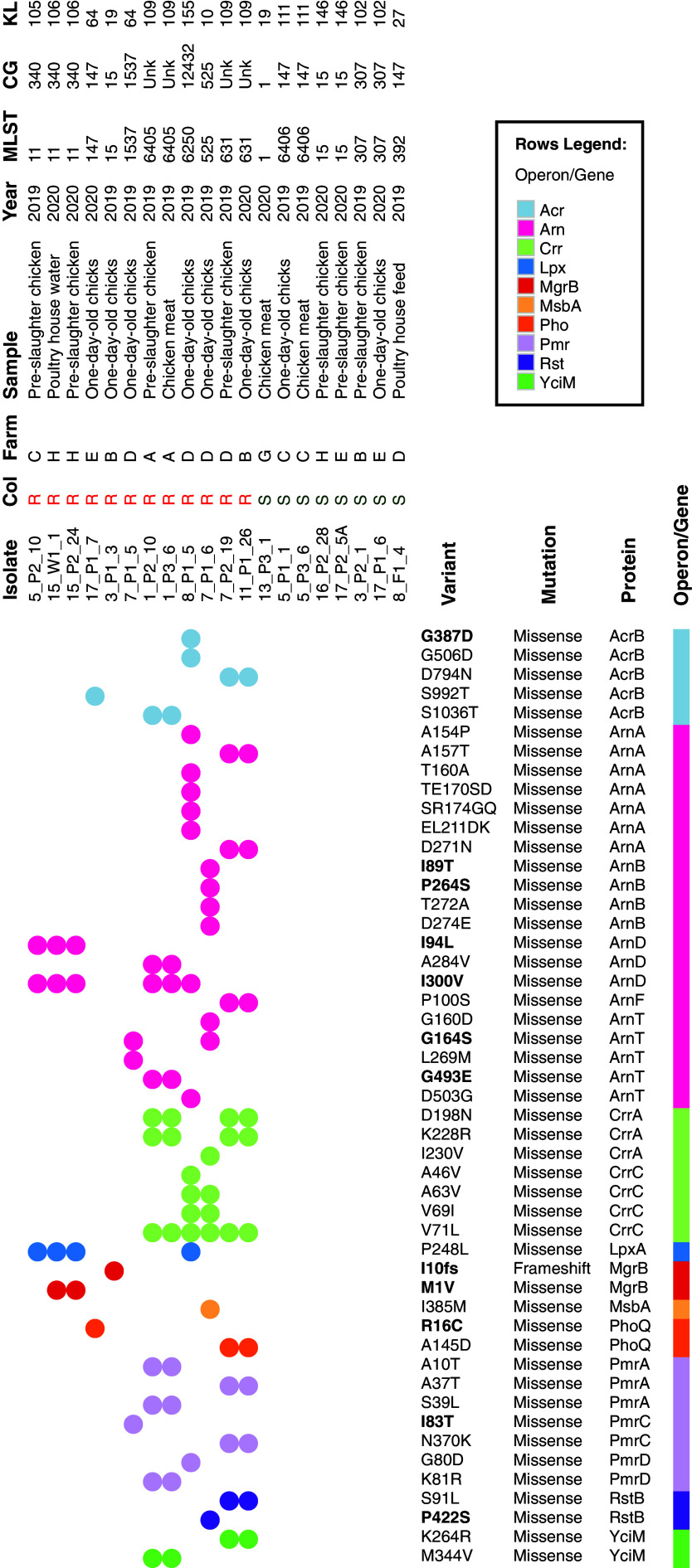
Heat map, generated using the Morpheus online web server (https://software.broadinstitute.org/morpheus/), representing the distribution of distinct mutations among the sequenced colistin-resistant Klebsiella pneumoniae genomes. Colored circles indicate the presence of a specific mutation, while each color represents the genes that are part of the same operon. Variants shown in bold were supported by the available literature as present in ColR-Kp and/or were predicted as deleterious by PROVEAN. Klebsiella pneumoniae isolates were grouped as colistin susceptible (S) when the MIC was ≤2 μg/mL or as colistin resistant (R) when the MIC was ≥2 μg/mL. When required, figures were minimally edited manually using Adobe Illustrator v25.3.1. Detailed resistance mechanisms: *acrB*, efflux pump; *arnABDFT*, lipid A modification with l-Ara4N addition; *crrA*, lipid A modification by upregulation of *pmrAB*/activation of the glycosyltransferase; *crrC,* connector protein; *lpxA*, inactivation of lipid A biosynthesis abolishing LPS synthesis; *mgrB*, inactivation of negative feedback regulator of the PhoP/PhoQ system; *msbA*, ABC transporter of lipid A; *phoQ* and *pmrA*, activation of LPS-modifying operation in the two-component systems; *pmrC*, lipid A modification with phosphoethanolamine; *pmrD*, connector protein; *rstB*, sensor protein; *yciM*, regulation of LPS biosynthesis. A, alanine; C, cysteine; D, aspartate; E, glutamate; G, glycine; I, isoleucine; K, lysine; L, leucine; M, methionine (start codon); N, asparagine; P, proline; R, arginine; S, serine; T, threonine; V, valine; Unk, unknown.

WGS revealed a high load and diversity in antibiotic resistance and metal tolerance genes in comparison to virulence genes (Fig. 5; Table S3). All isolates carried the chromosomal *mrkABCDFHIJ* cluster encoding type 3 fimbriae, whereas only ST15 isolates carried the virulence accessory genes *kfuABC* (ferric uptake system) and the *kpiABCDEFG* genes (pilus system). Regarding the genomic analysis of colistin resistance, we detected 96 different chromosomal mutations (mostly missense; *n* = 94/96) in 72% of the genes (*n* = 23/32) encoding proteins previously implicated in colistin resistance compared with the reference strain K. pneumoniae MGH 78578 (described in detail in Fig. 7; see also Fig. S1). From these, 52 distinct mutations were present in all ColR-Kp isolates (9 lineages) across five operons/genes associated with colistin resistance mechanisms such as modifications of lipopolysaccharide (LPS)/lipid A (*arnABDFT*, *crrAC*, *phoQ*, *pmrACD*, *mgrB*), overexpression of efflux pumps (*acrB*), LPS loss (*lpxA*, *msbA*) or biosynthesis (*yciM*), and regulation (*rstB*) (Fig. 7). Diverse mutations implicated in colistin resistance were detected in seven of those genes (*acrB*, *arnB*, *arnD*, *arnT*, *mgrB*, *phoQ*, and *pmrC*), varying accordingly with the clone (Fig. 7). All but one of the isolates accumulated mutations in different gene clusters associated with colistin resistance (ranging from two to seven).

In addition to colistin resistance, we detected diverse acquired genes (*n* = 36) encoding resistance to seven different antibiotic classes, with 85% of genomes (*n* = 16/20) carrying genes conferring resistance to ≥4 classes but differing between lineages (0 to 6). The most frequent classes and genes conferring resistance to antibiotics were aminoglycosides (*strA*/*strB*), sulfonamides (*sul1/sul2*), tetracyclines [*tet*(A)/*tet*(D)], trimethoprim (*dfrA*), and phenicols (*catA* and *cmlA*) (Fig. 5; Table S3). Also, analysis of the genetic background of resistance to ciprofloxacin showed chromosomal mutations in the quinolone resistance determining region (QRDR) of topoisomerase genes *gyrA* (S83I, S87A, and S83F) and *parC* (S80I) (*gyrA* and *parC*, 70%, *n* = 14/20) and variable plasmid-mediated quinolone resistance (PMQR) genes *qnrB* (*n* = 11) and *qnrS* (*n* = 6) (Fig. 5). Extended-spectrum cephalosporin gene *bla*_CTX-M-15_ was detected in only one ST307 isolate. Moreover, diverse acquired metal tolerance gene clusters encoding copper/silver (*pco/sil*), arsenic (*ars*), mercury (*mer*), and/or tellurite (*ter*) tolerance were detected in all but four of the genomes (Fig. 5). The *sil* and *pco* cluster (75%, *n* = 15/20) was frequently associated with operons *ars* (73%, *n* = 11/15), *mer* (67%, *n* = 10/15), and/or *ter* (40%, *n* = 6/15) (Fig. 5). Cooccurrence of antibiotic resistance genes and copper tolerance genes was detected in 75% (*n* = 15/20) of the genomes. Genomes carried an average of 5 plasmids (1 to 9 per genome) and a total of 18 different well-defined plasmid incompatibility groups (1 to 7 replicon types per isolate). The most common were IncFIB_K_ (80%; *n* = 16/20), followed by diverse IncFII_K_ (70%; *n* = 14/20) and IncR (70%; *n* = 14/20), most often in variable combinations with FIA, other FIB types (FIB_pNDM-MAR_, FIB_pCAV1099_, FIB_pKPHS1_), and/or HI1B (Fig. 5). Col plasmids were also frequent (65%; *n* = 12/20 genomes), while other classical incompatibility groups were rare (IncHI2, IncX, IncQ, and IncM). Copper tolerance genes *pco* and *sil* were located mainly in IncFIB_K_ and IncFII_K_ plasmids (73%; *n* = 11/15; ~80 to 270 kb) of different IncFII_K_ groups by pMLST (2, 4, 5, 7, 8, and 21-like) along with variable antibiotic resistance genes [*aac(3)-IVa*, *aph(4)-Ia*, *aadA2*, *strA*-*strB*, *qnrB1*, *catA2*, *sul1*, *sul2*, *tetA*, *tetD*, *dfrA12*, *dfrA14*, *bla*_TEM_, and *bla*_CTX-M-15_] (Fig. 5). These plasmids carrying *pco* and *sil* were similar to others (MOB-recon; mash distance, 0.0012 to 0.0309) described in humans in multiple countries, occasionally carrying *bla*_CTX-M-15_, *bla*_DHA-1_, and/or *qnr* genes (Table S4).

## DISCUSSION

This study first showed the absence of *mcr* but a high occurrence and diversity of colistin-resistant (*mcr*-negative) K. pneumoniae isolates after >2 years of colistin withdrawal in intensive chicken farms, independent of the type of Cu supplementation used in the feed formulation. Furthermore, we demonstrated the persistence of particular K. pneumoniae clones throughout the whole poultry production chain and suggested poultry as a potential foodborne/environmental source of K. pneumoniae lineages with clinical relevance.

The high rates of intensively raised flocks positive for K. pneumoniae in the early and preslaughter stages, together with the detection of K. pneumoniae with or without standard fecal indicators in feed or water samples (e.g., Escherichia coli and/or *Enterococcus* detected in 9 of 14 water samples from all farms) (data not shown), suggest diverse contamination events (e.g., hatchery farm, poultry house cleanliness and biosecurity level, water, feed, inanimate surfaces, and/or human handlers) occurring early and frequently along the production chain (34, 35). Environmental factors (e.g., type of litter, temperature, relative humidity, daily cycles, moment of sampling/age, season, and vacancy period) or the use of antimicrobial compounds (e.g., disinfectants, antibiotics, coccidiostats, or metals) are also known to have an impact on the composition of poultry gut microbiota (35–37). These factors could justify the differences found in fecal K. pneumoniae occurrence rates at the farm level in this study (82%, ranging from 25 to 100%) and compared to another recent study also using SCAi medium for bacterial recovery in chicken farms (26%) (38). However, despite the high rate of fecal samples carrying K. pneumoniae, resistant or not to colistin, our data revealed few positive chicken meat batches (17%), which is far below what was reported in studies from the United States (47%) and the European Union (60%) (39, 40). Besides, Salmonella was not detected in any sample (data not shown). These results demonstrate reduced meat cross-contamination and the effectiveness of sanitary measures during animal transport and at the slaughterhouse, thus reducing the consumer’s risk of exposure (12, 37, 41).

Recent studies suggest that banning colistin in food animal production has had an encouraging outcome by limiting *mcr* spread (11, 12). Our results confirm this trend in poultry production by the absence of *mcr* in farms and meat isolates, although a high rate of samples carried ColR-Kp associated with a high diversity of chromosomal mutations, independently of the feed type used. This suggests the circulation of diverse colistin-resistant genotypes responding differently to the colistin ban. We detected mutations, alone or in combination, in genes known to be involved in lipid A modifications and colistin resistance phenotypes (*arnABDFT*, *phoQ*, *pmrACD*, and *mgrB*), as described previously (42–50). However, a high variety of other nondescribed mutations were detected, supporting the urgent need for reliable genotypic-phenotypic correlations to explain colistin resistance mechanisms (51). Such a variety and frequency of chromosomal mutations in ColR-Kp isolates detected in different studies and environments (26, 43, 50, 51) suggest that they could play a role in adaptive features other than colistin resistance (e.g., *mgrB* inactivation in environmental K. pneumoniae survival and transmission or *phoPQ* or *pmrB* mutations in chlorhexidine tolerance) (52–54). Also, some of these mutations do not confer significant biological cost (55, 56), which may explain the occurrence of colistin-resistant *mcr*-negative K. pneumoniae isolates long after colistin withdrawal. Finally, other factors contributing to coselection (e.g., other antibiotics and growth promoters such as Cu) or maintenance (continuous contamination sources) of chromosomally mediated colistin-resistant strains in the livestock sector (26) could not be excluded.

In the context of antibiotic/colistin reduction and replacement, the use of diverse antimicrobial compounds may be expanded, making it difficult to understand the complexity of possible coselection events of the MDR strains. Some recent studies performed after colistin withdrawal revealed an increase in clinically relevant antibiotic resistance genes (e.g., *bla*_CTX-M_ and *bla*_NDM_) among food animal isolates (57, 58). In this study, these genes were not detected, but we showed high rates of resistance to multiple antibiotics in K. pneumoniae isolates from poultry, independently of the sample selection strategy (with or without colistin supplementation) or between sample groups (poultry life stages and feed type). Resistance rates were higher for antibiotics (e.g., ciprofloxacin and tetracycline) frequently used for therapeutics at poultry farms (12, 59), in contrast with other studies in poultry or other nonclinical environments (38, 60) where resistance rates were low. Such differences in the available studies could reflect local variation in the usage of several antibiotic classes, the diversity of routes for K. pneumoniae dissemination within and beyond the production environment, and the circulating clonal lineages (38, 60).

Other largely used feed ingredients with antimicrobial activity like metals/copper may also contribute to changes in poultry microbiota and/or even potentiate Cu-tolerant and MDR strain coselection (19, 20, 24), a factor unexplored for K. pneumoniae. Recent studies revealed correlations between metal environmental pollution (e.g., Cu at poultry farm samples) and an increase in antibiotic resistance genes (61, 62). We have also detected the plasmid-acquired copper tolerance *pco* plus *sil* cluster in isolates dispersed in all farms and in most of the flocks. Furthermore, we describe for the first time in K. pneumoniae a correlation between the presence of *pco* and *sil* genes and the Cu tolerance phenotype (MIC ≥ 16 mM in anaerobiosis). These data suggest the adaptability of this species to stressful/unfavorable farming environments, as previously detected for other zoonotic bacteria such as the emergent MDR clones of Salmonella (63). According to our data, the feeding regime (ITMF or OTMF) does not seem to contribute to the expansion of MDR or ColR-Kp. These data suggest that the similar whole-farm environment (e.g., litter quality, ventilation system, and temperature control) and management practices (e.g., sanitation and disinfection protocols, waste, and water control) overlap with the feeding regime. Thus, studies evaluating environmental factors (e.g., pH) (64) and subinhibitory concentrations of heavy metals to maintain antibiotic-resistant bacteria in poultry production and other related environments are urgent (61, 65, 66).

It is of note that metal tolerance operons (*pco* plus *sil*) were mainly located in multireplicon F type (FII_K_ plus FIB_K_) plasmids carrying genes encoding resistance to several classes of antibiotics, including the critical extended-spectrum cephalosporins and/or fluoroquinolones, supporting the potential for diverse coselection events (67, 68). These mosaic F type plasmids are common in K. pneumoniae populations from different sources (68–73), suggesting a major role in both dissemination and persistence of antibiotic resistance and metal tolerance genes and K. pneumoniae adaptation to different niches. Besides, the similarity between plasmid backbones identified in poultry isolates and those described in K. pneumoniae collections from humans (see Tables S3 and S4 in the supplemental material) (72) suggests a common pool of shared plasmids between humans and eventually different animal species (70), which deserves to be further explored with comprehensive comparative plasmidome analysis.

This is the first study tracking K. pneumoniae throughout the whole poultry production chain, enabling the identification of multiple transmission routes in flocks and the farm environment. The identification of closely related MDR K. pneumoniae isolates (e.g., ST11-KL106, ST15-KL146, ST631-KL109, ST6405-KL109, and ST6406/CG147-KL111) in multiple poultry stages and environments in the same or different farms suggests that food sector efforts should be made in improving the sanitary measures in the poultry houses, poultry workers, and the environment beyond (treatment of wastewaters and manure). The persistence of MDR K. pneumoniae clones in the poultry chain, which is according to previous studies conducted on farms (34, 35, 38) or at slaughterhouses (41), suggests the presence of adaptive environmental features other than antimicrobial resistance (e.g., biofilm formation). Most available studies assessing K. pneumoniae sources from a One Health perspective have included retail foods, livestock farms, or wastewater, while the poultry chain has been greatly understudied (27, 38–40, 59, 60, 74, 75). Despite the high diversity found, we highlight the commonality of KL types (KL64, KL102, KL105, and KL106) and lineages (ST15-KL19, ST15-KL146, and ST392-KL27) identified between poultry and human clinical isolates (this study) (28, 76). Although the immunogenicity of different KL types is not well understood, particular capsular types have been shown to influence the selection of certain K. pneumoniae lineages (77), which might provide an advantage regarding host immunity (78, 79). Finally, although human contamination cannot be excluded, our data suggest poultry as a reservoir and source of globally dispersed and human clinically relevant K. pneumoniae lineages with a potential risk for transmission to humans and other ecosystems (animals and the environment).

### Conclusions.

This study constitutes the first comprehensive analysis of K. pneumoniae from a poultry farm-to-fork perspective. Our data revealed, independently of the Cu feed type, a high rate of chicken fecal samples carrying a high diversity of K. pneumoniae isolates, including MDR, Cu-tolerant, and colistin-resistant/*mcr*-negative strains, long after colistin withdrawal. Furthermore, we propose that poultry may serve as a reservoir and source of human clinically relevant lineages and mosaic F type plasmids (with antibiotic resistance and metal tolerance genes), which could pose a potential risk of human exposure through the food chain (e.g., poultry carcasses and occupational exposure) and/or environmental release (e.g., farm or slaughterhouse wastewaters). Therefore, it is crucial to further explore the drivers contributing to the circulation and persistence of MDR K. pneumoniae in poultry production. This will help to identify novel mitigation strategies addressing producers’ efforts, consumer concerns, and the EU farm-to-fork goals in the fight against antimicrobial resistance and toward improving food safety and environmental sustainability.

## MATERIALS AND METHODS

### Sampling strategy at the chicken farm and slaughterhouse processing plant.

Our pilot study involved seven Portuguese intensive farming-based chicken farms with similar conventional indoor and floor-raised production systems, comprising all the key practices and requirements in compliance with EU legislation, as indicated by the operators (80). To ensure proper management, the farms were selected based on having grow-out poultry houses with similar conditions and individual feed silos, allowing for the division of each flock upon arrival (1-day-old chicks) into two groups, each receiving a different feed type (OTMF or ITMF). In all farms, colistin had been banned since January 2018, while copper was routinely used as an additive in poultry feed. The current inorganic formulation feed (ITMF) was supplemented with inorganic sources of Cu, and the organic formulation (OTMF) was supplemented with organic forms, both previously evidenced as capable of maintaining broiler performance under commercial conditions (23). Both mineral supplements were added to the same commercial starter and grower feeds adapted to different periods of the broiler’s life, with copper concentrations decreasing ≈42% in OTMF versus ITMF feeds (both far below the maximum dose of 25 mg/kg of Cu, according to EU Regulation 2018/1039).

**FIG 8:**
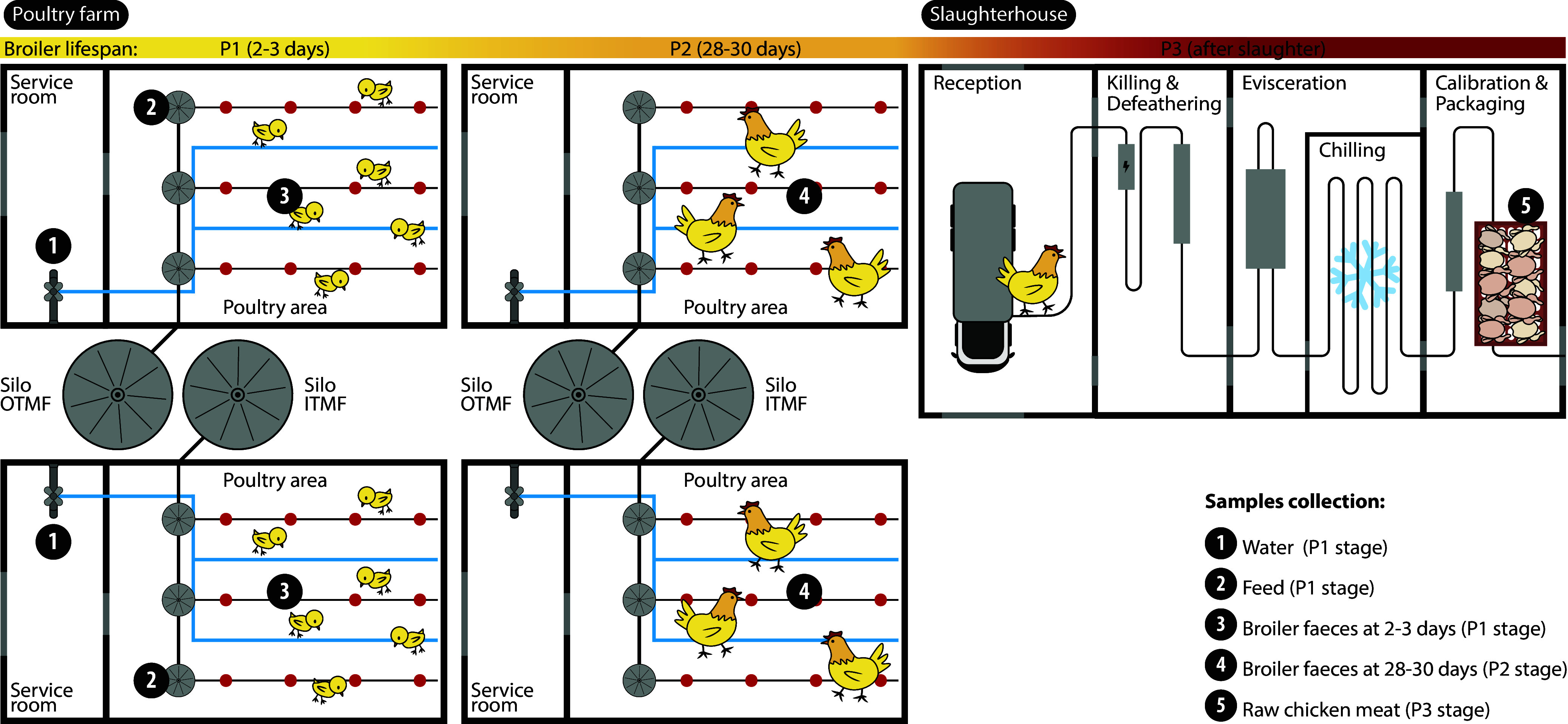
Sampling strategy at the chicken farm and slaughterhouse processing plant. Sample collection points are indicated by numbers.

Eighteen chicken flocks (10,000 to 64,000 animals from each house; Ross 308 strain) fed with ITMF (*n* = 9) or OTMF (*n* = 9) were sampled at each of the seven farms between October 2019 and November 2020 (two farms were sampled twice in different seasons). Personal protective equipment such as gloves, boots, and coveralls were used. Pooled fresh chicken feces (≈50 g; total *n* = 34) were collected with sterile spatulas/shovels from the floor by walking in a zig-zag pattern in two separated poultry houses (ITMF or OTMF) in each farm. The collection period was at 2 to 3 days of life (P1 stage; *n* = 18 samples) and the day before slaughter when the chickens were 28 to 30 days old (P2 stage; *n* = 16 samples; 2 samples missing during 2020 COVID-19 lockdown). Environmental samples, including feed (≈50 g collected at the silo that supplied the feeding lines; *n* = 18) and water (≈1 L collected at the faucet that supplied the drinking water lines; *n* = 14), were also collected inside grow-out houses at each farm at the P1 stage (Fig. 8).

Raw chicken meat samples (*n* = 18 batches; recovered after slaughter and air chilling) of the same flocks were collected after slaughter (P3 stage) in the poultry production slaughterhouses, immediately before distribution for retail sale. Each meat sample included approximately 50 g of neck skin cut with a sterile scalpel from a pool of 10 carcasses from the same batch (each batch corresponded to one flock from the same farm slaughtered at the same time) (Fig. 8).

All previous solid and water samples were collected in sterile plastic bags or containers, transported at 4°C, and processed on the same day at the laboratory. Subsequent sample processing was performed by culture approaches, as described in the following sections.

### Screening of K. pneumoniae and colistin-resistant K. pneumoniae.

K. pneumoniae was selectively recovered, directly from the sample and after enrichment, in the Simmons citrate agar plates with 1% inositol (SCAi), a medium that selectively favors the growth of Klebsiella in potential Escherichia coli-rich samples (see Fig. S2 in the supplemental material). A common initial step consisted of weighing a portion of 25 g of tested solid samples (P1, P2, and P3 stages) or the 0.45-μm filter from filtration of 500 mL of water samples (P1 stage), suspended in 225 mL of buffered peptone water (BPW) supplemented with 3.5 mg/L of colistin. The direct culture method included spreading an aliquot of 100 μL of the BPW plus colistin after 1 h at room temperature (resuscitation step) on SCAi supplemented or not with colistin (3.5 mg/L). The enrichment approach involved the same procedure, but after a previous incubation of BPW plus colistin at 37°C for 16 to 18 h. All the SCAi plates were incubated at 37°C for 48 h. One to five colonies of each presumptive morphotype were selected for identification. Isolate identification was performed by matrix-assisted laser desorption ionization–time of flight mass spectrometry (MALDI-TOF MS) (MALDI-TOF Vitek MS, bioMérieux, France) and by PCR for K. pneumoniae (81). In all the identified isolates, screening of colistin resistance genes (*mcr-1* to *mcr-5* and *mcr-6* to *mcr-9*) was assessed by two multiplex PCRs, as previously reported (82, 83). The MIC for colistin was determined by the reference broth microdilution (84). An estimation (in CFU per gram) of colistin-resistant K. pneumoniae in the poultry samples directly plated on SCAi plus colistin (see the procedure described above) was performed after counting typical Klebsiella colonies, species identification, and determination of colistin MIC.

### Phenotypic and genotypic characterization of K. pneumoniae.

Relatedness between isolates from different samples was inferred by Fourier transform infrared (FT-IR) spectroscopy with attenuated total reflectance (ATR) using a PerkinElmer Spectrum Two instrument. After growth under standardized culture conditions (37°C, 18 h), a colony was directly deposited on the ATR accessory of the FT-IR instrument and air dried. Spectra were acquired under standardized conditions (4,000 to 600 cm^−1^, 4 cm^−1^ resolution, and 16 scan coadditions). The region corresponding to polysaccharides (1,200 to 900 cm^−1^) was then compared between each other and with those from an in-house spectral database (allowing identification of 30 KL type(s) from well-characterized international K. pneumoniae clones), as previously described (76, 85). FT-IR-based assignments were confirmed using PCR of the *wzi* gene and further sequencing at Eurofins Genomics (https://www.eurofinsgenomics.eu/) to infer the KL type(s) at BIGSdb (https://bigsdb.pasteur.fr/klebsiella/) (86).

Antibiotic susceptibility profiles were determined by disc diffusion using 14 antibiotics (amoxicillin plus clavulanic acid, 30 μg; amikacin, 30 μg; aztreonam, 30 μg; cefepime, 30 μg; cefotaxime, 5 μg; cefoxitin, 30 μg; ceftazidime, 10 μg; chloramphenicol, 30 μg; ciprofloxacin, 5 μg; gentamicin, 10 μg; meropenem, 10 μg; sulfamethoxazole, 300 μg; tetracycline, 30 μg; trimethoprim, 5 μg). The interpretation was performed using the guidelines of the European Committee of Antimicrobial Susceptibility Testing (EUCAST) (87) and, when this was not possible, by the Clinical and Laboratory Standards Institute (CLSI) guidelines (88). Escherichia coli ATCC 25922 was used as the control strain. MDR was considered when the isolates were resistant to three or more antibiotics of different families (in addition to ampicillin, to which all K. pneumoniae are intrinsically resistant).

All the isolates were screened for the *silA* and *pcoD* Cu tolerance genes by PCR, as previously reported (63). Copper susceptibility was studied in representative isolates (representing the diverse farms, flocks, feeds, and genomic backgrounds) by the agar dilution method (MIC_CuSO4_, 0.5 to 36 mM; anaerobiosis), as previously published (63).

### WGS and comparative analysis.

Representative isolates (*n* = 20) of different farms, sources, time spans, and clones/KL type(s) were selected for WGS. The DNA was extracted with the Wizard genomic DNA purification kit (Promega Corporation, Madison, WI), with the final concentration measured with a Qubit 3.0 fluorometer (Invitrogen, Thermo Fisher Scientific, USA) and sequenced with an Illumina NovaSeq 6000 S4 PE150 XP system (Illumina, San Diego, CA) at Eurofins Genomics (https://eurofinsgenomics.eu/). Raw read quality control was assessed with default parameters using FastQC v0.11.9 (89) and MultiQC v1.12 (90). Good-quality raw reads were then *de novo* assembled using SPAdes v3.15.3 (91) with “–isolate” and “–cov-cutoff auto” flag options. We then used QUAST v5.0.2 (92) and CheckM v1.0.18 (93) within KBase (https://www.kbase.us/) to assess the quality and completeness of the assemblies.

The assemblies were annotated using the RAST server (94). Genome assemblies were then uploaded to Pathogenwatch v2.3.1 (https://pathogen.watch/) to determine species, capsular polysaccharide (K) and lipopolysaccharide O locus types and serotypes (29), MLSTs (95), and cgMLSTs. Pathogenwatch uses the calculated pairwise SNP distances between the genomes based on a concatenated alignment of 1,972 core genes to infer a neighbor-joining tree for phylogenetic analysis (96). Closely related genomes and the associated metadata (country, source of isolation, and collection date) were retrieved from all public genomes available at Pathogenwatch, after cgMLST single linkage clustering and selection of those with less than 10 allele differences (threshold = 10). Neighbor-joining trees were edited using iToL (97).

Snippy v3.2-dev (https://github.com/tseemann/snippy) was used to identify substitutions (SNPs) and insertions/deletions (indels) in genes (*n* = 32) putatively associated with colistin resistance (43, 51, 98), by aligning the raw read data from each isolate against the reference genome Klebsiella pneumoniae MGH 78578 (GenBank accession number CP000647). All gene mutations were further manually confirmed in the assembled genomes using the Geneious Prime 2023.0.1 software (http://www.geneious.com/). The deleterious effect of detected mutations on protein function was further assessed using the PROVEAN protein variation effect analyzer (99) or data from previous publications. We used the standard PROVEAN scores of less than or equal to −2.5 for a deleterious effect and greater than −2.5 for a neutral effect on protein function.

Kleborate v2.2.0 (100), integrated into Pathogenwatch, was used for the prediction of antibiotic resistance determinants (acquired and chromosomal mutations) and virulence traits (yersiniabactin YbST, colibactin CbST, aerobactin AbST, salmochelin SmST, and regulators of mucoid phenotype RmpA and RmpA2). ABRicate v1.0.1 (https://github.com/tseemann/abricate) with in-house databases was used to detect additional K. pneumoniae virulence genes from BIGSdb-Pasteur (https://bigsdb.pasteur.fr/klebsiella/), the chaperone-usher pilus system (*kpiABCDEFG*) (101), and relevant metal tolerance genes (*cusABFCRS*, *arsBCR*, *arsR1H-arsD1A1A2-arsCBA3D2R2*, *pcoGE1ABCDRSE2*, *silESRCFBAGP*, *merRTPCADE*, and *terZABCDEF-terWY1XY2* operons). AMRFinderPlus v3.10.30 (102) was used for the determination of antibiotic-resistant novel alleles.

In all 20 WGS-selected isolates, plasmid replicon typing was determined using PlasmidFinder (103, 104) and pMLST v2.0 (104) from the Centre for Genomic and Epidemiology (http://www.genomicepidemiology.org). IncFII_K_ plasmids were further characterized according to reference 105 (https://pubmlst.org/organisms/plasmid-mlst). To confirm the location of metal tolerance genes and reconstruct putative plasmids based on draft assemblies, we used the MOB-recon tool v3.0.0 from the MOB-suite package (106, 107). The metal tolerance genes were considered part of a specific plasmid when identified by MOB-recon or when located on the same contig as the replicon/incompatibility determinant.

### Statistical analysis.

Differences in occurrence, antimicrobial resistance, copper tolerance, and bacterial count among K. pneumoniae isolates and flocks fed ITMF and OTMF as well as among P1, P2, and P3 stages were analyzed by Fisher’s exact and Wilcoxon matched-pairs signed rank tests (α = 0.05) using Prism software, version 8.1.1 (GraphPad).

### Data availability.

The data for this study have been deposited in the European Nucleotide Archive (https://www.ebi.ac.uk/ena) under BioProject accession number PRJEB60418.
